# Atezolizumab-induced Autoimmune Diabetes in a Patient with Metastatic Breast Cancer: A Case Report

**DOI:** 10.5811/cpcem.2021.2.51508

**Published:** 2021-05-10

**Authors:** Robert Clontz, Duc M. Dang, Michelle A. Hieger, Brent A. Becker

**Affiliations:** Wellspan York Hospital, Department of Emergency Medicine, York, Pennsylvania

**Keywords:** Atezolizumab, autoimmune diabetes mellitus, immune checkpoint inhibitors, case report

## Abstract

**Introduction:**

Immune checkpoint inhibitors (ICI) are a class of immunotherapy drugs used increasingly in the treatment of multiple types of cancer. Major side effects include immune-related adverse effects, potentially resulting in damage to normal tissue across multiple different organ systems.

**Case Report:**

A 74-year-old woman with a history of triple negative metastatic breast cancer treated with the ICI atezolizumab presented with new-onset autoimmune diabetes in diabetic ketoacidosis. She required fluid resuscitation, insulin infusion, vasopressors, and initial hospitalization in the intensive care unit. The patient was subsequently discharged on bolus dose insulin and remained an insulin-dependent diabetic at three-month follow-up.

**Conclusion:**

Autoimmune diabetes is a rare, but life-threatening, adverse event associated with ICIs such as atezolizumab. To our knowledge this is the only case report of atezolizumab causing autoimmune diabetes in the setting of metastatic breast cancer. As ICIs become more common in the treatment of cancer, emergency physicians should remain vigilant for the various immune-mediated complications associated with this class of immunotherapy drugs.

## INTRODUCTION

Immune checkpoint inhibitors (ICI) are a class of immunotherapy drugs used increasingly in the treatment of multiple types of cancer. Immune checkpoint inhibitors function by removing inhibitory signals for T-cells and facilitating antitumor activity; however, this can also result in autoimmune-mediated damage to normal tissue. Known as immune-related adverse effects (irAE), these side effects most often affect the skin and gastrointestinal, and endocrine systems. Hypophysitis and thyroid dysfunction are the most common endocrine abnormalities.^1^,^2^

Atezolizumab is a monoclonal ICI, first approved by the US Food and Drug Administration (FDA) in 2016 for the treatment of metastatic urothelial cancer. It is currently used in multiple other malignancies, including urothelial carcinoma and triple negative breast cancer.^1^,^3^ We describe a case of atezolizumab-induced, new-onset diabetes presenting with diabetic ketoacidosis (DKA) in a patient with metastatic breast cancer.

## CASE REPORT

A 74-year-old woman presented to the emergency department with two days of nausea, vomiting, dyspnea, lightheadedness, and malaise. The patient had been diagnosed with invasive ductal carcinoma of the right breast 12 months prior to presentation. The malignancy was estrogen receptor, progesterone receptor, and human epidermal growth factor receptor 2 negative with known metastasis to the bone. After initial treatment with doxorubicin hydrochloride, cyclophosphamide and paclitaxel, the patient was transitioned to atezolizumab and paclitaxel combination therapy. She had completed 14 cycles over the preceding 52 weeks, and oncology reported her disease to be well controlled and stable. She carried a prior diagnosis of paroxysmal atrial fibrillation managed with diltiazem, metoprolol and warfarin.

The patient had no history of diabetes or insulin resistance and had previously demonstrated consistently normal serum glucose levels. There was no preceding starvation, insulin use, thromboembolic event, or ischemic process. She had been started on low-dose steroid of prednisone 5 milligrams (mg) daily three months prior for autoimmune-mediated arthritis and chemotherapy-induced neuropathy of her left lower extremity. Her non-fasting blood glucose level two months prior to presentation on routine blood work was 135 mg/deciliter (dL) (reference range 70 – 139 mg/dL).

On initial assessment, her vital signs were temperature 36.3º C, blood pressure 126/78 millimeters of mercury (mm Hg), heart rate 123 beats per minute, respiratory rate 24 breaths per minute, and oxygen saturation 96% on room air. She had atrial fibrillation with rapid ventricular response. Physical exam was significant for Kussmaul respirations, bibasilar rales, and mild subcostal retractions. Her body mass index was 39.4 kilograms per meter squared (kg/m^2^) (18.5 – 24.9 kg/m^2^).

Initial laboratory results are shown in the Table. She had marked hyperglycemia with an elevated anion gap acidosis, ketonuria, evidence of urinary tract infection, and supratherapeutic anticoagulation. Electrocardiography showed atrial fibrillation with rapid ventricular rate and no evidence of acute ischemic changes. Based on history, physical, and laboratory data it was determined that her presentation was most consistent with new-onset diabetes mellitus with DKA. Her case was further complicated by severe sepsis secondary to a urinary tract infection and atrial fibrillation with rapid ventricular response. The patient was treated with sodium chloride 0.9% at 150 milliliters (mL) per hour and an infusion of regular insulin (250 units in 250 mL of 0.9% sodium chloride) at 0.1 units/kg per hour. Her urinary tract infection was treated empirically with intravenous cefepime 1 gram and she received two 50 mL boluses of 8.4% sodium bicarbonate for severe metabolic acidosis.

The patient was persistently hypotensive with a blood pressure of 95/49 mm Hg following fluid resuscitation. She was subsequently started on norepinephrine 4 mg in 250 mL premix at 0.01 micrograms/kg per minute and transferred to the medical intensive care unit (ICU) for management of DKA and septic shock. Her urine culture later revealed greater than 100,000 colony-forming units/mL *Escherichia coli* at 48 hours. After three days in the ICU, the patient was transitioned to the general medical floor on a regimen of insulin glargine 30 units every morning and insulin lispro 7 units three times daily with meals.

During hospital admission, her C-peptide level was 0.10 nanograms/mL (ng/mL) (reference range 0.80–3.85 ng/mL). Glutamic acid decarboxylase antibodies were less than 5 international units per milliliter (IU/mL (<5 IU/mL). The diagnosis of new-onset diabetes was deemed autoimmune in nature and attributed to her use of atezolizumab over the prior year. She was discharged on hospital day eight on insulin lispro and insulin detemir and remained an insulin-dependent diabetic at three-month follow-up.

**CPC-EM Capsule**

What do we already know about this clinical entity?

*Immune checkpoint inhibitors are associated with rare yet potentially fatal immune-related adverse effects and can damage almost any organ system.*

What makes this presentation of disease reportable?

*To our knowledge this is the only case report of atezolizumab causing autoimmune diabetes in the setting of triple negative metastatic breast cancer.*

What is the major learning point?

*When treating cancer patients in the ED, it is crucial to determine the cancer diagnosis and treatment history including past and present types of therapeutics.*

How might this improve emergency medicine practice?

*Early recognition and diagnosis of immune-related adverse effects by emergency physicians can help provide timely acute management and improve patient outcomes*.

## DISCUSSION

We present a rare case of atezolizumab-induced autoimmune diabetes mellitus in a patient treated for metastatic breast cancer. While cases of ICI-induced autoimmune DKA have been previously reported related to the treatment of urothelial, skin, lung, and renal cancer, no prior case has been described in the setting of metastatic breast cancer treatment.^1^

Atezolizumab is a humanized monoclonal antibody to programmed death-ligand 1 (PD-L1) and is classified as an ICI. Atezolizumab is FDA approved for the treatment of various types of cancer, including metastatic non-small cell lung cancer, locally advanced or metastatic urothelial carcinoma, and triple-negative breast cancer in patients who experience disease progression after platinum-based chemotherapy. The recommended dose is 840 mg administered as an intravenous infusion every three weeks until disease progression or unacceptable toxicity is noted.^4^

Atezolizumab functions by inhibiting the binding of programmed cell death 1 (PD-1), which is expressed on T cells, to PD-L1, which is expressed on antigen-presenting cells and tumor cells (Figure, A). This inhibition allows the T cells to activate and proliferate against tumor cells. Unfortunately, pancreatic islet cells also express PD-L1. Atezolizumab can inhibit the binding of PD-1 to PD-L1 and activate the T cell against the pancreatic islet cells. Subsequent destruction of pancreatic islet cells results in autoimmune-induced diabetes (Figure, B).

The onset of ICI-mediated autoimmune diabetes has been shown to range from a single dose in the first week to 228 weeks, with a median onset of 20 weeks, after drug initiation.^3^,^5^–^7^ In this case, the time to onset was 52 weeks. The treatment of ICI-induced diabetes is similar to the treatment of new-onset diabetes of other causes. In our patient, DKA was identified and an insulin infusion was initiated and continued until the patient’s anion gap normalized. She was then switched to subcutaneous short- and long-acting insulin therapy. In ICI-induced diabetes, immunosuppression with corticosteroids can be attempted, but may be futile given that typically 80–95% of the insulin-producing pancreatic beta cells will have been permanently destroyed.^2^ This is in contrast to most other irAE where glucocorticoids are considered first-line therapy.^3^

In the majority of cases involving irAE secondary to ICI therapy, the emergency physician should discuss with oncology whether urgent initiation of glucocorticoids is indicated. Most patients can continue taking the ICI after stabilization of glucose levels because the majority of insulin-producing pancreatic cells have already been eliminated.^2^ The ICI-induced endocrine injury is typically permanent. To date, there have been no reported cases of diabetes remission with the cessation of ICIs.^5^

In addition to diabetes, there are a multitude of other immune-mediated complications associated with ICIs. A meta-analysis of anti-cytotoxic T-lymphocyte-associated protein 4 (CTLA-4) antibodies found the overall incidence of adverse events to be 72% with 24% involving high-grade complications.^8^ Such adverse events included skin lesions (rash, pruritus, and vitiligo), colitis, hepatitis, hypophysitis, thyroiditis, sarcoidosis, uveitis, Guillain-Barré syndrome, immune-mediated cytopenia, and polymyalgia rheumatica.^8^ Another study examining all three major classes of ICIs (CTLA-4, PD-1, and PDL-1) found 32.7% of emergency department patients on an ICI were diagnosed with an immune-mediated toxicity. Colitis (38.8%), hepatitis (19.4%), and pneumonitis (14.3%) were the most common immune-mediated toxicities. Of the 300 patients included in the study, DKA remained a rare presentation (0.3%).^9^ The incidence of thyroid disorders (hypothyroidism, thyrotoxicosis, painless thyroiditis, or thyroid storm) is estimated to be approximately 10% in patients treated with a single agent anti-PD-1/PD-L1.^10^

## CONCLUSION

Autoimmune diabetes is a rare but significant potential side effect of immune checkpoint inhibitor therapy. Emergency physicians should be aware of this and other immune-mediated complications in patients receiving ICI immunotherapy. Emergency physicians are likely to encounter more patients on ICIs and should routinely consider diabetes, diabetic ketoacidosis, and other acute conditions related to underlying immune-related adverse effects.

## Figures and Tables

**Figure:**
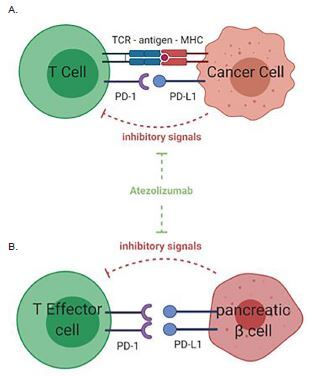
**(A)** Mechanism of anti-PD-L1, atezolizumab, on T-cell response to cancer cell. PD-L1 is expressed on cancer cells and inhibits T-cell activation when binding to the PD-1 receptor on T-cell surface. Anti-PD-1/PD-L1 treatment with atezolizumab leads to the inhibition of this inhibitory pathway and subsequent enhanced T-cell activity against tumor cells. **(B)** Mechanism of action of anti-PD-L1 agent on immune tolerance in the pancreas. PD-1/PD-L1 interaction between effector T cells and pancreatic insulin-secreting β cells inhibits T-cell activation against the pancreas. The anti-PD-L1 agent, atezolizumab, blocks the PD-1/PD-L1 interaction, leading to activation of self-reactive T cells and destruction of the pancreatic islet cells of Langerhans. This results in immunotherapy-induced autoimmune diabetes. Figures created with BioRender.com (Toronto, ON, Canada). *MHC*, major histocompatibility complex; *TCR*, T-cell receptor; *PD-L1*, programmed death – ligand 1; *PD-1*, programmed death – 1.

**Table:** Initial laboratory values of a patient with hyperglycemia, elevated anion gap metabolic acidosis, and evidence of urinary tract infection.

Laboratory Name (reference range with units)	Patient’s Results
Basic Metabolic Panel	
Sodium (135–145 mmol/L)	127
Potassium (3.5–5.3 mmol/L)	5.7
Chloride (98–107 mmol/L)	91
Carbon dioxide (21–31 mmol/L)	6
Blood urea nitrogen (7–25 mg/dL)	35
Creatinine (0.06–1.2 mg/dL)	1.39
Glucose (70–99 mg/dL)	845
Calcium (8.6–10.3 mg/dL)	9.0
Estimated glomerular filtration rate (>90 mL/min/1.73m^2^)	37.4
Anion gap (3–11 mmol/L)	30
Complete Blood Count	
White blood cell count (4–11 K/mcL)	17.4
Hemoglobin (11.5–15.5 g/dL)	13.5
Hematocrit (34.5–47.0%)	42.6
Platelets (140–400 K/mcL)	465
Absolute neutrophils (1.7–7.8 K/mcL)	13.81
Coagulation Studies	
Prothrombin time (9.4–12.4 seconds)	46.2
International normalized ratio (0.8–1.2)	4.6
Other Laboratory Values	
Venous pH (7.35–7.45)	7.06
Beta hydroxybutyrate (0.0–0.3 mmol/L)	12.3
Lactic acid (0.5–2.0 mmol/L)	2.6
Hemoglobin A1c (<5.7%)	7.1
Urinalysis	
Urine color (yellow, light yellow, dark yellow)	Yellow
Urine clarity (clear)	Cloudy
Leukocyte esterase (negative)	Trace
Nitrite (negative)	Negative
Urine glucose (negative mg/dL)	>1000
Urine ketones (negative mg/dL)	≥ 80
Urine blood (negative)	Moderate
Urine red blood cells (0–23 mcL)	14
Urine white blood cells (0–28 mcL)	396
Urine squamous epithelial (0–31 mcL)	5
Urine bacteria (0–358 mcL)	184

*mmol/L*, millimole per liter; *mg/dL*, milligrams per deciliter; *mL/min/1.73m^2^*, milliliters per minute per 1.73 meters squared; *K/mcL*, thousands per microliter; *g/dL*, grams per deciliter; *mcL*, microliter.
